# Lung Microbiome in Asthma: Current Perspectives

**DOI:** 10.3390/jcm8111967

**Published:** 2019-11-14

**Authors:** Konstantinos Loverdos, Georgios Bellos, Louiza Kokolatou, Ioannis Vasileiadis, Evangelos Giamarellos, Matteo Pecchiari, Nikolaos Koulouris, Antonia Koutsoukou, Nikoletta Rovina

**Affiliations:** 1 ICU, 1st Department of Pulmonary Medicine, “Sotiria” Hospital; Athens School of Medicine, National and Kapodistrian University of Athens, 11527 Athens, Greece; kloverdos@yahoo.com (K.L.); ioannisvmed@yahoo.gr (I.V.); koulnik@med.uoa.gr (N.K.); koutsoukou@yahoo.gr (A.K.); 2 Koropi Academic Health Care Center, Koropi, 19400 Attica, Greece; geobellos1010@yahoo.gr (G.B.); louikok@gmail.com (L.K.); 3 4th Department of Internal Medicine, Attikon University Hospital, 12462 Athens, Greece; egiamarel@med.uoa.gr; 4 Dipartimento di Fisiopatologia e dei Trapianti, Università degli Studi di Milano, 20122 Milan, Italy; matteo.pecchiari@unimi.it

**Keywords:** microbiome, pathogenesis, inflammation, immune responses, asthma

## Abstract

A growing body of evidence implicates the human microbiome as a potentially influential player actively engaged in shaping the pathogenetic processes underlying the endotypes and phenotypes of chronic respiratory diseases, particularly of the airways. In this article, we specifically review current evidence on the characteristics of lung microbiome, and specifically the bacteriome, the modes of interaction between lung microbiota and host immune system, the role of the “lung–gut axis”, and the functional effects thereof on asthma pathogenesis. We also attempt to explore the possibilities of therapeutic manipulation of the microbiome, aiming at the establishment of asthma prevention strategies and the optimization of asthma treatment.

## 1. Introduction

Asthma is the most common chronic respiratory disease, affecting more than 300 million people of all ages worldwide and killing about 250,000 of them each year [1], posing a substantial socioeconomic burden, especially in low- and middle-income countries. Asthma is a multifactorial and heterogeneous disease, comprising several different disease “phenotypes” and “endotypes” [2,3,4]. The current approach acknowledging that different phenotypes may share a common endotype and vice versa and, more important, that the disease phenotype may change over time has boosted our understanding on asthma pathogenesis and facilitated the development of novel targeted biological therapies, especially where they are most needed—that is severe corticosteroid-insensitive asthma [5]. However, while highly effective biologics are now available, and several more are in the pipeline for severe uncontrolled asthmatics with the Th2-high endotype [6], an almost-empty therapeutic arsenal is the case for those with the Th2-low endotype. Moreover, given the fact that Th2 inflammatory markers are absent in up to 50% of asthmatic patients (although an even higher corresponding percentage of 76% was reported in a very recent randomized controlled trial of patients with mild persistent asthma) [7,8], it is clear that further research is urgently needed to shed light on the biological pathways leading to non-eosinophilic asthma and, thus, promote the discovery of new treatment strategies for this large group of patients.

A growing body of evidence implicates the human microbiome as a potentially influential player that is actively engaged in shaping the pathogenetic processes underlying the aforementioned and other unresolved issues both in asthma [9,10] and in the other chronic respiratory diseases, particularly of the airways [11,12,13]. In contrast to earlier beliefs, a well-developed metabolically active microbial community, termed lung microbiota, resides in the lower respiratory tract of healthy humans. The entire habitat, including the microorganisms (bacteria, archaea, lower and higher eukaryotes, and viruses), their genomes (i.e., genes), and the surrounding environmental conditions are defined as the lung microbiome [14]. The latter is involved in a constant cross-talk with the host, and the same applies for the other bacterial communities residing in and on the human body, as well, with the most prominent being the gut microbiota, forming the “gut–lung axis” [15]. The diverse mechanisms mediating this cross-talk are now being gradually recognized. Under normal conditions; the interaction between the microbiota and the host confers mutual benefits for both (“symbiosis”). However, we are becoming increasingly cognizant of the fact that lung microbiota composition and diversity are affected in disease (including asthma) and that these changes can be translated in altered host immune responses, influencing asthma susceptibility, phenotype, exacerbation pattern, and treatment responsiveness (“dysbiosis” instead of “symbiosis”). 

In this article, we review current evidence on the characteristics of lung microbiome, the modes of interaction between lung microbiota and host immune system, the role of the “lung–gut axis” and the functional effects thereof on asthma pathogenesis. We also attempt to explore the possibilities of therapeutic manipulation of the microbiome, aiming at the establishment of asthma prevention strategies and the optimization of asthma treatment. 

## 2. Microbiome

### 2.1. Historical Perspectives

The perception of the existence of bacteria inhabiting certain parts of the human body without causing disease is not new. For decades, diverse microbial genera were isolated after in vitro cultivation of biological samples collected from healthy individuals. However, the sensitivity of these traditional culture-dependent microbiological methods was severely limited. For instance, in an early study, it was estimated that only 24% of the entire microbiota present in an adult male fecal sample could be recovered by cultivation [16]. Using a variety of media and incubation methods, the corresponding sensitivity of in vitro cultivation for bacterial species identification in bronchoalveolar lavage samples of healthy individuals was substantially higher (61%), but it was still limited [17]. However, the exact role and teleology of this “normal (micro) flora” remained largely unknown, although it was postulated that changes in its composition could be detrimental, as in the case of antibiotic-induced *Clostridium difficile* overgrowth in the large intestine of patients with pseudomembranous colitis [18]. The advent of novel molecular techniques for microbiological profiling, with the most important being the implementation of polymerase chain reaction (PCR) amplification and sequencing of the highly specific and ubiquitous among bacteria 16S-rRNA gene, led to a tremendous progress in our understanding of the extent, diversity, composition, and location of the sum of microbial communities living inside or on the human organism (now termed microbiota instead of normal flora) [19]. Large-scale research collaborations, such as the two phases of the Human Microbiome Program (HMP), funded by the US National Institutes of Health (NIH), and the Metagenomics of the Human Intestinal Tract (MetaHIT) project, funded by the European Community, established enormous reference databases of human microbiota genomes and metagenomes, after analyzing dozens of thousands of samples derived from 48 primary sites (mostly feces) in hundreds of healthy individuals and patients with specific conditions or disorders [20,21,22,23,24]. Based on these advances, it is now estimated that human microbiome consists of approximately 3.8 × 10^13^ bacteria, probably marginally outnumbering human cells (3 × 10^13^ for the standard age and somatotype) [25,26]. As expected, the microbial community residing in the gut is the most abundant, comprising slightly more than 1000 bacterial species [24,27]. These commensal bacteria harbor about 3.3 million genes, surpassing in number the genes contained in the host genome by approximately 150 times [24]. It soon became evident that this incredibly rich microbial ecosystem could not be uninvolved in the biological processes underlying health and disease. Further evolution in molecular biology, namely the emergence of “-omics” technologies (genomics, transcriptomics, proteomics, and metabolomics), have now enabled the investigation of the functional effects of human microbiome by detecting and studying the functional genes encoded by the microbial community and their products (proteins, metabolites etc.) [19].

Amplicon-based sequencing of marker genes, such as 16S rRNA, is a powerful tool for assessing and comparing the structure of microbial communities at a high phylogenetic resolution. Because 16S rRNA sequencing is more cost-effective than whole-metagenome shotgun sequencing, marker gene analysis is frequently used for broad studies that involve a large number of different samples. With the expanded use of 16S rRNA sequencing for resident microbiota recognition on different human surfaces, organs such as the lungs, the stomach, and the uterus, previously considered sterile based on culture-dependent studies, were shown to host a substantial microbial burden under normal conditions. These findings gave birth to the notion of lung microbiome and primed tenacious research endeavors for its characterization.

### 2.2. The Lung Microbiome in Health

#### 2.2.1. The Early Life Shaping

**Figure 1:**
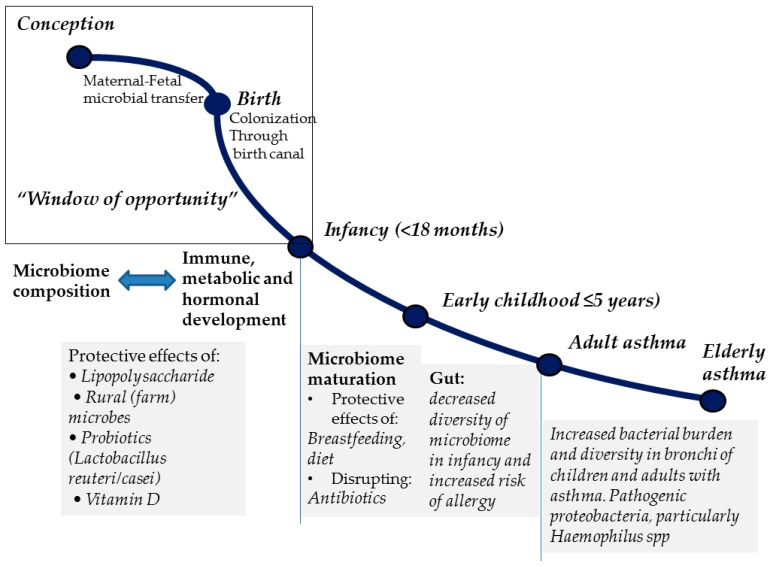
The natural history of microbiome development.

Although not specifically studied in humans, the development of the lung microbiome probably adheres to that of the rest of the human body microbial ecosystem. The exact starting time point for the bacterial colonization of the human body cannot be accurately determined. Until recently, amniotic fluid, which fills fetal lungs prenatally, was considered sterile. This historical belief was challenged by the discovery of bacterial DNA in amniotic fluid and placental samples [28], which may be suggestive of a prenatal initiation of lung microbial colonization and development, although the actual existence of an amniotic fluid microbiome remains controversial [29] and its potential significance vastly unknown. Detectable microbial communities in multiple body sites have been identified in newborns as early as <5 min after delivery, and their synthesis initially resembles the maternal vagina or skin microbiota composition, depending on the mode of delivery (vaginal or caesarian section) [30]. This premature microbiome has been shown to change in composition and diversity and mature functionally during the first two to three years of life, after which it gradually stabilizes to a pattern closely matching that of adults [31,32,33]. This early life microbiota instability, probably in parallel with the concurrent immune system immaturity, is believed to render microbiome particularly susceptible to the influence of diverse environmental factors, including diet (e.g., breastfeeding), day care, crowding, and antibiotic use [34,35], which ultimately shape the structure of the adult human microbiome and, presumably, confer predisposition or resistance to disease (“window of opportunity” theory) [36] (see Figure 1). A similar trajectory of diversity and composition changes with increasing age has recently been described in the lung microbiota of mice [37].

#### 2.2.2. The Immigration/Elimination Balance 

**Figure 2:**
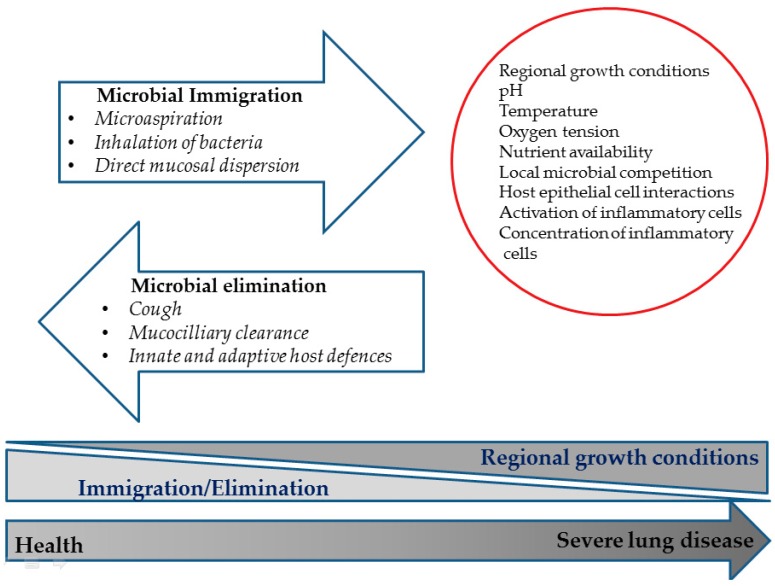
Factors determining the immigration/elimination balance.

The synthesis of any bacterial (or other living organism) community at any given time is dictated by the interaction between three factors: (1) immigration, (2) elimination, and (3) local reproduction of community members (see Figure 2). 

With regard to lung microbiome, immigration mostly originates from subclinical micro-aspiration from the upper respiratory tract (URT), although other sources, including the inspired air (which carries approximately 10^5^–10^6^ bacteria/m^3^) [38], and the upper gastrointestinal tract via aspiration of gastric contents [39], may also make minor contributions. The elimination of lung microbial community members can be assumed to be mediated by the complete armory of lung defense mechanisms, including cough reflex, mucociliary clearance, and innate and adaptive immunity [40], and, thus, to depend on its effectiveness. In health, the balance between dispersal of microbes from the URT and eradication of lung microbial community members via local defense mechanisms is considered the major determinant of lung microbiome characteristics, whereas local bacterial reproduction most probably plays a rather minor role. The URT as the major source of lung microbiome is strongly supported by data showing a close resemblance between upper and lower respiratory tract (LRT) microbiome composition in healthy individuals [41,42,43]. Further corroboration of these results comes from the study of Venkataraman et al., demonstrating that lung microbiome composition in healthy individuals could be best attributed to neutral dispersal of microbes from the oropharynx rather than active local bacterial selection in the lungs [16]. Even more so, in a study investigating the possibility of spatially determined intrapulmonary discrepancies in the characteristics of lung microbiome, Dickson et al. showed that microbial richness and Firmicutes phylum abundance in the right upper lobe was more similar to those of the supraglottic region compared with the other lung lobes sampled, in which microbiome is practically identical [44]. Given the closer vicinity of the right upper lobe to the URT, these findings suggest that not only is the LRT microbiome directly related to that of the URT, but also this association is probably inversely proportional to the distance from the oropharynx (i.e., the more proximal to the oropharynx, the closer the microbiome resemblance). Conversely, if intrapulmonary bacterial growth was a major determinant of lung microbiome synthesis, significant intra-subject variations in the microbiome characteristics of different lobes should have been expected, given the well-established between-lobes disparities in local growth conditions (e.g., oxygen tension, pH, temperature) [45]. This was not the case in the study of Dickson et al, in which all parts of the lung located distally from the URT, irrespective of exact lobe, had practically identical microbiome [44].

#### 2.2.3. Composition and Structural Features

In contrast with the microbial communities residing in other parts of the human body, our knowledge on normal lung microbiome features, in terms of its development, composition (particularly at the genus and species levels), functional effects, and their determinants remains largely incomplete. To some extent, this reflects sampling difficulties, resulting in most relevant studies suffering from small-size limitations and lack of longitudinal data [13]. 

Lung microbiota is a relatively small bacterial community. Based on the findings of studies applying 16S rRNA sequencing in endobronchial brushing (EB) samples from healthy and diseased individuals, it is estimated that there are on average 10^3^–10^5^ bacterial genomes (or 16S copies) per cm^2^ of bronchial tissue sampled, although with significant inter-subject variability [41,46]. Comparatively, colon microbiota, which is the most abundant microbial ecosystem in the human body, comprises up to 10^11^ CFU/gr of luminal content [47]. In a study evaluating the associations between the diverse microbial communities of the aero-digestive tract, bacterial density in bronchoalveolar lavage (BAL) fluid was found to be 100- to 1000-fold and 10- to 100-fold lower than in oral washes and gastric aspirates, respectively, of the same healthy subjects [43]. Sequencing of these 16S rRNA genes and comparison with established microbial genomic databases have led to the identification of 38 bacteria phyla, with 303 [48], or even more [49], genera residing in the human lung. However, these are far from equally represented with the top six phyla and the top 25 genera accounting for 86% and 65%, respectively, of all sequences identified [48]. Specifically, *Bacteroidetes* and *Firmicutes* are the most abundant phyla in the lung microbiota of healthy humans, followed by *Proteobacteria* and, to a lesser extent, *Actinobacteria* and *Fusobacteria* [41,43,46,48,50]. At the genus level, *Prevotella*, *Veillonella*, and *Streptococcus* are generally considered the most dominant taxa [41,42,46,51], although there is substantial variation in the relevant abundance of lung commensal microbes between studies and *Neisseria, Haemophilus, Fusobacterium*, or other genera (e.g., *Actinomyces, Porphyromonas,* and *Lactobacillus*) are occasionally found in comparable counts [42,46,47,48,49,52]. Although these discrepancies are probably, at least in part, due to the small sizes of the relevant studies, the presence of considerable inter-subject variability in lung microbiota composition has been documented in healthy individuals [44,52], so that, to date, it is not possible to define a typical (“normal”) lung microbiome. 

### 2.3. Potential Effects of Sampling Methods on the Assessment of Lung Microbiome Structure 

The study of lung microbiota parameters requires bronchoscopy for BAL or EB samples acquisition. Induced sputum analysis may be a less invasive alternative, although differences in bacterial composition of sputum samples compared with those retrieved by bronchoscopic techniques, as a result of contamination by the rich oral/oropharyngeal microbiota, have been well documented [49]. Moreover, there is a certain degree of uncertainty surrounding putative effects of the specific bronchoscopic technique used for sampling on the characteristics of the derived microbiota. Denner et al. reported significant discrepancies in the extent, diversity, and relative affluence of the retrieved microbial communities between BAL and EB bronchoscopic sampling, the latter being associated with a denser and more diverse microbiome [48]. An earlier study comparing the microbial communities sampled by multiple respiratory tract sites of healthy individuals, demonstrated that the bacterial population of the left lower lobar bronchus retrieved by EB was generally larger than the populations of the right middle lobe segmental bronchi, which were sampled by consecutive BALs, although no differences were found in the corresponding bacterial communities’ composition [41]. Other researchers have ruled out bronchoscopic-technique-specific effects on the lung microbiota synthesis after applying both BAL and EB in contralateral lobes of healthy volunteers [44]. Although the reported discrepancies could be merely the result of differences in the nature of the samples (and in the modes of their acquisition), along with small study sizes, they might also represent a shift in the microbiota characteristics of the small peripheral airways and the lung parenchyma (sampled by BAL) compared with the more proximal bronchi (sampled by EB). Until further research addresses these issues and establishes a standardized technique for lung microbiota derivation, sampling methods should be taken into account when designing or evaluating the results of human lung microbiome studies.

### 2.4. Spatial Discrepancies in the Structure of Lung Microbiome

Some degree of spatially dependent intra-subject variability in lung microbiota features has been demonstrated, although this is substantially more limited than the aforementioned between-subjects variability [44]. Specifically, it has been shown that the right upper lobe microbial community richness, composition, and variation are all significantly different from those of more distal parts of the lungs (left upper lobe, right middle lobe, and lingula) of the same healthy individuals and more closely resemble the upper respiratory tract microbiota [44]. Finally, it should be mentioned that, in contrast with the gut microbiome which has been shown to present significant geographical variation possibly associated with the regional lifestyle [32], there are no data suggesting a similar trend in lung microbiome. Despite the lack of direct comparisons between populations from different parts of the world, no dissimilarities have been found in the lung microbiome of HMP initiative participants from eight different US cities [42] and, to date, the relatively few studies conducted in non-Western populations do not seem to yield different results from the majority of lung microbiome studies, which have generally been confined to the Western world [53,54]. 

#### Cross-Talk between Lung Microbiota and the Host

**Figure 3:**
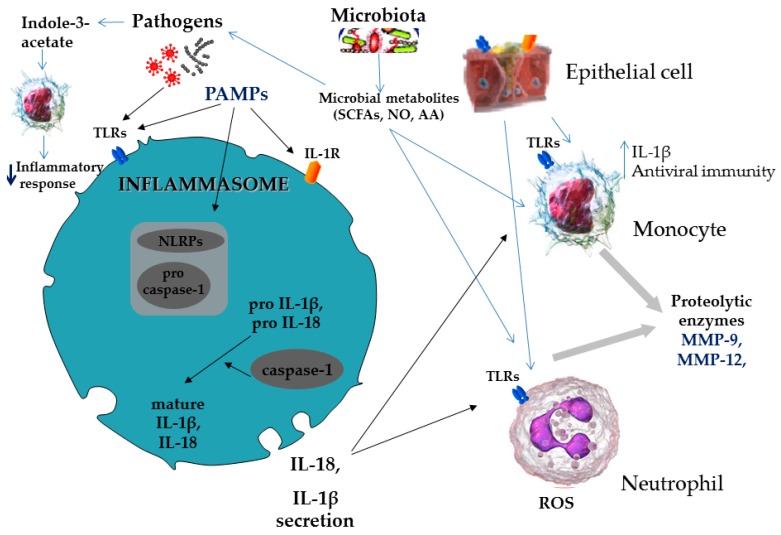
Respiratory microbiota produce metabolites (SCFAs, NO, or nitrite, aromatic amino acids), which influence host immune activity. Inflammasome-associated pro-cytokines can be produced and activated by a family of receptors which detect the presence of pathogens through PAMPs. SCFA: short chain fatty acids; NO: nitric oxide; AAs: aromatic amino acids; TLR: Toll-like receptors; ROS: reactive oxygen species; PAMP: pathogen associated molecular patterns; MMP: matrix metalloproteinase; MDC: macrophage-derived chemokine; MIP1α: macrophage inflammatory protein 1α.

The host immune system is primarily responsible for the conduct of most of the host–microbiome interplay, and there is now a growing body of evidence establishing the presence of an active and multiform cross-talk between the lung microbiome and the host immune system [11]. Invading pathogens activate the inflammasomes (multi-meric protein complexes) both directly and indirectly [55], to produce inflammasome associated pro-cytokines (IL-18, IL-1β), after the recognition of the pathogens by a family of receptors through pathogen-associated molecular patterns (PAMPs) [56]. Structural components of the bacterial cells and LPS (a ubiquitous structural component of Gram-negative bacteria outer membrane) are ligands for the pattern recognition receptors (PRRs) expressed by the host antigen-presenting cells. Upon stimulation, PRRs (with Toll-like receptors (TLRs)-2 and -4 being the principle representatives) may trigger diverse cellular processes regulating immune responses in the lung. Importantly, these responses appear to be bacterial species- or genus-specific, underscoring the potentially significant effects of lung microbiome composition alterations on the host immune regulation (see Figure 3).

Interspecies differences in LPS structure are thought to account for the corresponding variations in TLR subtype specificity and lung inflammatory capacity [57,58]. The Bacteroides *Prevotella*, one of the most abundant genera in the healthy lung microbiome, appears to exhibit a TLR2-dependent low inflammatory potential, whereas the Proteobacteria *Haemophilus influenzae* and *Moraxella catarrhalis,* linked with lung microbiome alterations in asthma and COPD, induce severe TLR2 independent (and probably TLR4-dependent) lung inflammation and injury in mice [57]. Other potentially pathogenic Proteobacteria residing in the lung, such as *Pseudomonas aeruginosa*, *Stenotrophomonas maltophilia*, and *Burkholderia* spp, possess flagella, the major structural component of which flagellin is recognized by host TLR5, leading to the induction of pro-inflammatory mediators’ secretion [59]. Bacterial DNA may also stimulate host immune responses. This effect is mediated by the abundant in bacterial DNA sequences unmethylated CpG dinucleotides, which bind to the host TLR9, inducing an inflammatory response of the T-helper-1 (Th1) type [60]. Furthermore, all four major phyla of the lung microbiome (Bacteroidetes, Firmicutes, Proteobacteria, and Actinobacteria) have been shown to stimulate NOD2 receptors in vitro, and this effect is mediated by the bacterial cell wall component peptidoglycan [61]. 

Beyond structural microbial components, resident microbes-derived metabolites are also believed to be actively involved in the interplay between lung microbiome and the host immune system. Short-chain fatty acids (SCFAs) and amino acids metabolism products are the most extensively studied metabolites generated by human microbiota. SCFAs, such as butyrate, propionate, and acetate, are the main end-product of dietary fiber fermentation undertaken by the gastrointestinal tract microbiota. However, there are also data implying SCFAs production from lung microbiota, since active expression of bacterial genes associated with SCFAs metabolism has been described in bronchial brushings [62]. SCFAs have been implicated in numerous mechanisms promoting maintenance of homeostasis in health, including preservation of gut barrier integrity, control of appetite and energy intake, protection against autoimmunity and tumorigenesis in colon, and regulation of blood–brain barrier permeability, among others [63,64]. Importantly, they have also been shown to possess immunomodulatory properties [64,65]. The latter are mediated by G protein coupled receptors, particularly GPR41, GPR43, and GPR109a, which are all expressed on the surface of most types of inflammatory cells (macrophages/monocytes, dendritic cells, and neutrophils), as well as by direct inhibition of histone deacetylases (HDACs) [63,64,65], i.e., enzymes actively participating in post-translational modifications of the histones-DNA interaction inside the chromatin structure that regulates cell transcriptional activity and gene expression [66]. The principal immunomodulating effect of SCFAs appears to be the induction of differentiation and proliferation of extra-thymic regulatory T cells (Tregs) [67,68,69], a lymphocyte subset with established anti-inflammatory and anti-allergic effects [70,71]. Apart from the SCFAs, indole-3-acetate, a metabolite of the amino acid tryptophan produced by gut microbiota, was recently shown to attenuate LPS-induced pro-inflammatory cytokine and chemokine secretion in alveolar macrophages derived from smokers, and this observed anti-inflammatory action of indole-3-acetate was hypothesized to potentially mediate the effect of azithromycin on lowering exacerbation rates in COPD patients [72]. Indole-3-acetate and the other tryptophan metabolites are activators of the aryl hydrocarbon receptor [73], another well-known modulator of inflammation and immunity, with a proposed role in Treg generation [74].

We previously focused on microbiota-derived mediators engaged in shaping diverse aspects of host immune response. It must be realized that the opposite is most probably also valid. This means that numerous signaling molecules originating from host cells can be sensed by resident microbes and are capable of modifying the composition and diversity of lung microbiome. Indeed, there are data from several in vitro studies demonstrating potential influences of catecholamines and cytokines on the growth and virulence of various bacterial strains, perhaps with a species- or genus-specific manner [75,76,77,78,79]. This host-to-microbiome signaling pathway may be implicated in respiratory disease pathogenesis by contributing to lung microbiome alterations favoring the dominance of specific potentially pathogenic species. In this context, it has been shown that increased intra-alveolar levels of the catecholamines epinephrine and norepinephrine in BAL samples from lung-transplant recipients are significantly associated with both indices of acute infection and reduced lung microbiome diversity, characterized by the predominance of a single bacterial species, notably *Pseudomonas aeruginosa* [80].

### 2.5. Microbiome and Asthma Susceptibility

#### The “Gut–Lung Axis” and the Hygiene Hypothesis

**Figure 4:**
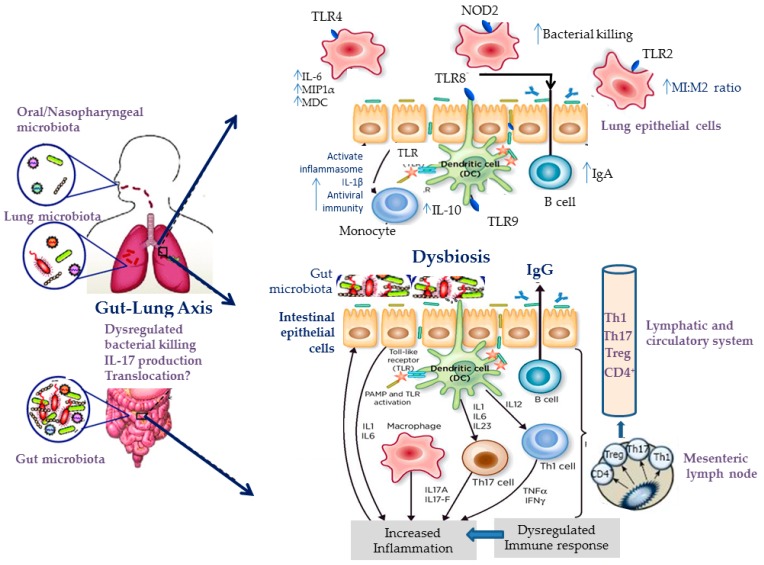
The “lung–gut” axis interactions with host immunity. TLR: Toll-like receptors; Treg: T regulatory cell; MDC: macrophage-derived chemokine; MIP1α: macrophage inflammatory protein 1α.

Accumulated evidence arising from both human and animal studies suggests that the development of allergic diseases, including asthma, may, in fact, be dependent on the bacterial communities residing in the gut. Gut microbiota actively interact with the host immune system via bacterial structural components and secreted metabolites, and these interactions possess the ability to modulate immune responses both locally in the gastrointestinal tract and systemically, influencing various distal sites, including the lungs. The term “gut–lung axis” has been coined to account for these effects of gut microbiota on lung immunity, both in health and in disease [81] (see Figure 4).

**Figure 5:**
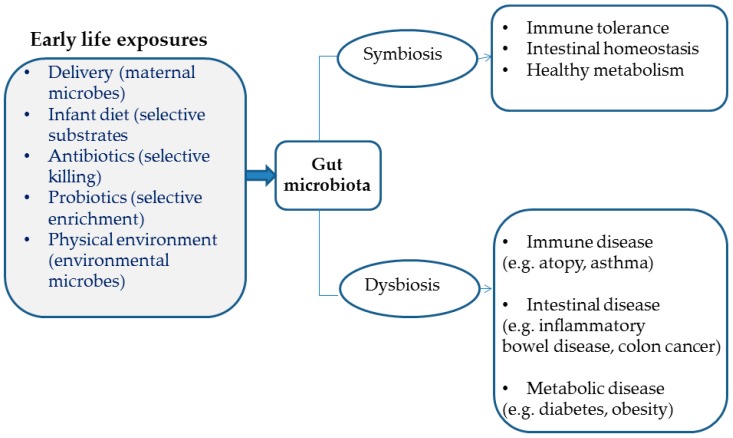
Early life exposures that may affect the symbiosis/dysbiosis balance and predispose to asthma.

The presumable involvement of resident microbiota in allergic asthma development is primarily supported by data showing an exaggerated Th2 immunity-driven airway inflammation in germ-free (GF) mice sensitized with ovalbumin compared with specific pathogen-free (SPF) counterparts, which can be abrogated when GF mice are recolonized by the commensal flora of the SPF animals prior to sensitization [82]. Likewise, gut microbiota disruption as a result of antibiotics administration promotes allergic airway disease in experimental murine asthma [83,84]. Importantly, antibiotic-induced long-term alterations in gut microbiota diversity and composition have also been observed in humans [85,86,87] and both early life and maternal antibiotic use have been associated with an increased risk for recurrent wheeze and asthma development in childhood [88,89]. Aside from antibiotic exposure, formula feeding [90,91] and Caesarian-section delivery [92,93] have been correlated both with differences in infant gut microbiota composition and with a heightened childhood asthma susceptibility compared with breastfeeding and vaginal delivery. Other exposures operating in early life, when the microbiome and the host immune system are both in the process of maturation, might also be relevant (see Figure 1 and Figure 5). For instance, living with dogs or cats as pets during the first year of life has been linked to a decreased prevalence of atopy at age six to seven [94], and dust from households with dogs has been shown to enrich cecal microbiota, together with downregulating Th2-mediated airway inflammation in a murine model of allergic sensitization [95]. Fujimura et al. [95] elegantly showed that exposure to pets may be associated with distinct gut microbiota composition characteristics conferring protection against airway allergen challenge [95]. Specifically, the cecal microbiome of mice exposed to dust derived from households with dogs was significantly enriched in Firmicutes compared with mice exposed to dust from residencies without pets, with *Lactobacillus johnsonii* being the most dominant taxon.

The relative microbial diversity of the environment and the level of exposure during the first years of life have also been highlighted as important factors influencing subsequent allergy and asthma susceptibility. Several studies from around the world have consistently demonstrated that children growing up in rural settings with high-level exposure to bacterially enriched farming environments presented significantly lower rates of atopic asthma compared with their non-farm peers [96]. In a landmark study published in 2016, 60 schoolchildren from two reproductively isolated US agricultural communities, the Amish and the Hutterites, sharing strong similarities in terms of genetic background and lifestyle, but with diametrically opposite farming practices, were examined [97]. The children of the Amish, who practice a traditional type of farming, presented a significantly lower rate of allergic sensitization compared to those of Hutterites, who use highly industrialized farming infrastructure, and no asthma cases were identified among Amish, in contrast to the six asthmatic Hutterite children. 

Short-chain fatty acids (SCFAs) have been proposed as pivotal mediators of the gut–lung interplay. Knockout mice lacking the *gpr43* receptor gene have been shown to present an exaggerated allergic airway inflammatory response upon ovalbumin challenge [98]. In a landmark study, Trompette et al. demonstrated that the dietary fiber content may influence the extent of allergic inflammatory changes in the lungs via SCFAs-mediated defects in DC activation, leading to an impaired Th2 cell differentiation [99]. Interestingly, they showed that high-fiber diet attenuated Th2-driven lung inflammation, as assessed by total and differential cell count in BAL fluid, Th2 cytokines mRNA levels in lung tissue, serum total IgE, metacholine challenge, and lung histological analysis. On the contrary, low-fiber diet aggravated allergic inflammation in the lungs. Furthermore, the administration of SCFAs to mice through drinking water led to an acceleration of Th2 inflammation resolution in propionate-supplemented wild-type mice compared with un-supplemented controls, which was abrogated in a separate group of GPR41-dedicient mice. These results indicate that circulating SCFAs (propionate in particular) produced by gut resident bacteria in direct proportion to the dietary fiber content may reduce susceptibility to allergic airway disease through the activation of the GRP41 receptor. 

### 2.6. The Lung Microbiome in Asthma 

During the last decade, an ever-growing number of studies have attempted to shed light on the features of lung microbiome in patients with asthma [46,48,50,62,100,101,102,103,104,105,106,107,108]; however, the small sample sizes, the lack of uniformity and standardization in the selection and processing of respiratory samples used for microbiome characterization in asthma, the scarcity of data on potential longitudinal changes in lung microbiome composition during the course of the disease and in association with treatment implementation, and finally, the absence of a clearly described ‘normal’ lung microbiome with which comparisons can be safely made pose limitations to the complete delineation and interpretation of lung microbiome in asthmatic patients. Despite these caveats, studies investigating the lung microbiome in asthma have been relatively successful in capturing microbiome alterations in a large part of the spectrum of disease severity and phenotypes, thus providing an initial premature understanding of a potential association between features of lung microbiome and specific disease characteristics.

#### Alterations of Lung Microbiome Structure in Asthma 

The most constant finding of lung microbiome studies in asthma is probably an observed increase in the relative abundance of the Proteobacteria phylum in asthmatic lungs [9,46,62,106,107,108,109]. At the genus level, this change is seemingly driven by a corresponding increase in the prevalence of *Haemophilus* and/or *Neisseria* [46,62,103,105,106,107], although other potentially pathogenic genera belonging to the Proteobacteria phylum, such as *Moraxella*, *Pseudomonas*, and members of the *Enterobacteriaceae* family, might also be involved [48,103,106,108]. It appears, though, that this Proteobacteria expansion is rather specific to non-severe asthma, as studies directly comparing non-severe with severe asthmatics have demonstrated distinct patterns of lung microbiome composition in these two groups, with Proteobacteria dominating in the lung microbiome of non-severe asthmatics, while other phyla, possibly Actinobacteria [108] or Firmicutes (mainly *Streptococci*) [106], are more prevalent in severe asthma. However, an increased relative abundance of certain Proteobacteria, such as the *Pseudomonadaceae* and *Enterobacteriaceae* (most notably *Klebsiella* spp), has also been reported in severe asthma patients [104,108]. 

Other taxa, e.g., the common respiratory commensals *Prevotella* and *Veillonella*, have generally been shown to be less common in the lung microbiome of patients with both severe and non-severe asthma [46,48,50,106], although these findings are not universal [62]. Overall, asthma-associated alterations in lung microbiome composition are significantly less firmly determined at the genus level compared with the phylum level, possibly reflecting, at least in part, the corresponding variations described in the lung microbiome composition of healthy individuals.

The relationship between asthma and lung microbial community diversity is even more controversial. Earlier studies in patients with mild asthma have reported an increased bacterial burden and diversity in induced sputa and EBs compared with healthy individuals [101,102]. In line with these observations, Durack et al. found a marginally increased phylogenetic diversity in EB samples retrieved from 42 asthmatic patients compared with 21 healthy controls [62]. Likewise, Sverrild et al. showed bacterial diversity augmentation in BAL fluid specimens from 23 patients with non-eosinophilic asthma [107]. On the contrary, other studies having included patients with more severe or corticosteroids refractory disease failed to replicate these findings and occasionally came up with opposite results [50,105,106,108]. 

### 2.7. The Potential Role of Lung Microbiome in Shaping Asthma Phenotypes and Endotypes

**Figure 6:**
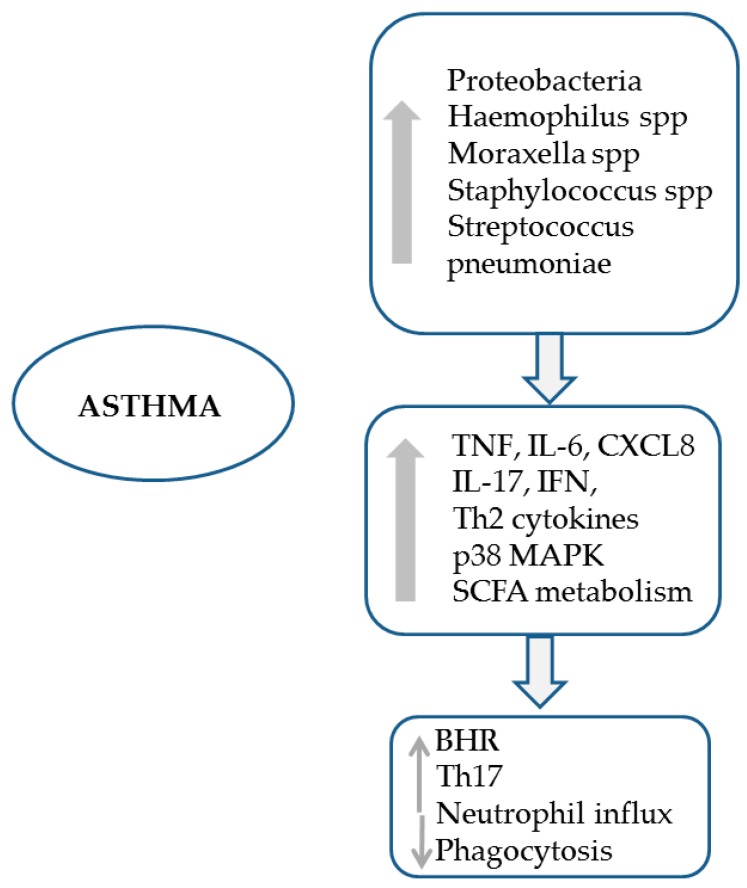
Implication of lung microbiome in asthma. TNF: Tumor necrosis factor; IL: Interleukin; IFN: Interferon; MAPK: Mitogen-activated protein kinase; SCFA: short chain fatty acid; BHR: bronchial hyperresponsiveness.

Several clinical, physiological, and inflammatory characteristics involved in the definition of asthma phenotypes (and, to a lesser extent, endotypes) [3] have been linked with features of lung microbiome (see Figure 6).

#### 2.7.1. Inflammatory Profile 

##### Eosinophilic Inflammation

Lung microbiota diversity and composition have been investigated in patients with both severe and non-severe eosinophilic asthma and compared with the corresponding features in non-eosinophilic asthma. In a cohort of mild corticosteroid (CS)-naïve asthma, Sverrild et al. reported increased α-diversity (a measure of bacterial taxa richness and evenness) and decreased β-diversity (an index of heterogeneity between bacterial communities) in the BAL microbiome of eosinophil-high asthmatic patients compared with eosinophil-low ones [107]. At the same time, various genera were significantly enriched (e.g., *Aeribacillus, Halomonas,* and *Sphingomonas*) or depleted (e.g., *Neisseria, Bacteroides,* and *Actinomyces*) in eosinophil-high as opposed to eosinophil-low asthma. On the contrary, in another cohort encompassing patients with both severe and non-severe asthma, the *Actinomycataceae* family members were significantly more abundant in eosinophilic compared with non-eosinophilic, asthma and their relative abundance was positively associated with the eosinophil count in sputa [108]. *Tropheryma whipplei* has also been identified as a prevalent member of the lung bacterial community in severe asthmatic patients of the eosinophilic inflammatory phenotype [105].

Instead of directly comparing lung microbiome composition in patients with eosinophilic and non-eosinophilic asthma, other groups have focused on the investigation of potential associations between features of microbiome and markers of eosinophilic airway inflammation. Eosinophil infiltration of bronchial tissue and eosinophil count in BAL fluid have been associated with lower total bacterial loads and diversity in EB samples from patients with mostly severe asthma [48,104]. Particularly, *Rickettsia* and certain Actinobacteria have been positively correlated with lung eosinophilia, whereas taxa negatively correlated mostly belong to Proteobacteria (including *Moraxellaceae*) and Firmicutes [48,104]. The bronchial epithelial cell expression of specific genes known to be involved in the Th2-mediated immune response (*CLCA1*, *SERPINB2* and *POSTN*) has also been examined in relation to lung microbiome composition [62,104]. These three gene expressions correlated negatively with total bacterial burden and relative abundance of certain taxa, including the previously mentioned *Moraxellaceae* [62,104].

Despite significant variability and, to some extent, rather contradictory results between studies, these data strongly support the existence of distinct features of lung microbiota burden, diversity, and composition in patients with the eosinophilic asthma subtype. Overall, it appears that both the eosinophilic inflammatory phenotype and the underlying Th2-high endotype correspond to lung microbial communities with comparatively low bacterial load, substantial diversity but limited heterogeneity, and possibly increased representation of Actinobacteria (perhaps most notably of the *Actinomyces* genus) and decreased representation of certain Proteobacteria (potentially including *Moraxella* sp) [110]. 

##### Neutrophilic Inflammation

Neutrophilic asthma, along with the paucigranulocytic inflammatory phenotype, belongs to the non-Th2 (or Th2-low) asthma endotype and Th1 and/or Th17 immune processes have been implicated in its pathogenesis [109,111], although therapeutic targeting of neither Th1- nor Th17-related cytokines, namely anti-TNFa [112] and anti-IL17A receptor agents [113], have proved effective. On the other hand, certain bacterial pathogens are established triggers of Th17-driven immune responses [114], and there are some data showing reduction of neutrophilic inflammatory markers (primarily IL-8) and quality-of-life improvement following treatment with clarithromycin in patients with severe refractory non-eosinophilic asthma [115]. These observations have drawn attention to the investigation of potential associations between lung microbiome and neutrophilic asthma in particular. In the most recent and largest to-date study of lung microbiome in asthma, Taylor et al. classified 167 patients with moderate-to-severe asthma in eosinophilic, neutrophilic, paucigranulocytic, and mixed granulocytic inflammatory phenotypes based on induced sputum differential cell count percentages and searched for interphenotype dissimilarities in sputum bacterial diversity and composition [110]. Although limited by the uneven distribution of participants across the different phenotypes (only 14 had neutrophilic asthma), the study provided evidence for a significantly decreased bacterial diversity, along with increased heterogeneity in patients with neutrophilic compared with both eosinophilic and paucigranulocytic asthma. This was accompanied by a strong inverse correlation between phylogenetic diversity and neutrophil percentage in sputum. *Haemophilus* and *Moraxella* genera were found enriched in neutrophilic asthma samples and significantly correlated with asthma inflammatory profile, with the opposite being the case for *Streptococcus*. In line with these results, Simpson et al. also demonstrated a decreased bacterial diversity in induced sputa collected from patients with severe neutrophilic compared with non-neutrophilic asthma in an earlier study [105]. Again, Proteobacteria, particularly *Haemophilus influenzae*, was the phylum dominating in the neutrophilic airway microbiota, with a relative depletion of Actinobacteria and Firmicutes. Similarly, others have shown positive correlations between *Moraxella catarrhalis*, *Haemophilus* sp, and *Streptococcus* sp total abundance and sputum neutrophils percentage and IL-8 concentration in severe asthma [103].

Although observational, and thus not establishing causality, these studies may support a case for lung microbiota dysbiosis in the pathogenesis of neutrophilic asthma. It appears that the LRT of asthmatic patients with predominantly neutrophilic airway inflammation harbors a relatively uniform microbiome, in which Proteobacteria, most prominently the potentially pathogenic *Haemophilus* and *Moraxella* genera, have outgrown the taxa normally over-distributed in the lung microbiota (e.g., Firmicutes). These gradually expanding new colonizers of the asthmatic airways may, in fact, constitute the triggering factor for the aberrant Th17 immune response, along with other inflammatory pathways activation, observed in neutrophilic asthma. Indeed, non-typeable *Haemophilus influenzae* intranasally administered at a sublethal dose has been shown to produce a robust Th17 response in the lungs of experimental mice [116] and, even more relevantly, infection with non-typeable *Haemophilus influenzae* may lead to reduced eosinophilic inflammation and increased neutrophilic infiltration of the airways in an IL-17-dependent manner in a murine model of allergic asthma [117]. Although the cause of Proteobacteria overgrowth in the LRT of certain asthmatics remains elusive and may be multifactorial (e.g., selective bacterial growth favored by structural changes and/or inflammatory mediators in the airways as a result of the underlying disease process), it must be noted that, as previously discussed, inhaled corticosteroid (ICS) treatment itself may promote Proteobacteria enrichment in the lung microbiota and, thus, contribute to the development of neutrophilic asthma [2]. Apparently, if this hypothetical association between respiratory dysbiosis and neutrophilic asthma pathogenesis stands true, strategies aiming at the manipulation of lung microbiome, possibly through Proteobacteria suppression and normal diversity restoration, may open up new avenues for the treatment of this, until now, untargeted asthma phenotype. Macrolides may be part of such strategies. 

#### 2.7.2. Corticosteroid Responsiveness

Several data support the correlation between lung microbiota burden and diversity and corticosteroid responsiveness. Goleva et al, in a study on CS sensitive and CS resistant asthmatics, demonstrated that, although bacterial composition both at the phylum and at the genus level was quite similar between the two groups overall, unique patterns of bacterial expansions were observed in the majority of patients with both CS-resistant and CS-sensitive asthma [50]. In CS-resistant asthmatics, the genera *Neisseria, Haemophilus,* and *Tropheryma* were abundant, while the genera *Pasteurella* and *Fusobacterium* were predominant in CS-sensitive asthma. Of note, CS-resistant patients had significantly higher levels of interleukin (IL)-8 mRNA in their BAL cells, implying that distinct lung microbiota profiles may be responsible for CS resistance in the neutrophilic asthma phenotype. To further validate their findings, the authors subsequently incubated alveolar macrophages isolated from BAL samples of asthmatic patients, with either *Haemophilus parainfluenzae* (a genus solely expanded in CS resistant patients) or *Prevotella melaninogenica*, and found a significantly reduced CS responsiveness in vitro following treatment with dexamethasone in cells cultured with *H. parainfluenzae,* but not *P*. *melaninogenica*. Durack et al. assessed bacterial composition in ten ICS responders and an equal number of ICS non-responders, both with mild ICS-naive asthma [62]. They reported significantly different microbiota profiles in the two groups, with responder’s lung microbiome synthesis sharing more resemblance with that of healthy controls. In particular, the *Microbacteriaceae* and *Pasteurellaceae* (including *Haemophilus*) families were found enriched in ICS non-responders and expansions of the *Streptococcaceae, Fusobacteriaceae,* and *Sphingomonodaceae* families were shown in responders. Interestingly, in the same study, lack of CS responsiveness was associated functionally with increased expression of bacterial genes involved in biodegradation pathways, which may explain resistance to CS treatment.

Finally, Huang et al. demonstrated a significant positive correlation between lung microbiota diversity and *FKBP5* gene expression, a marker of steroid response [104]. In terms of bacterial composition, increased *FKBP5* gene expression was associated mainly with Actinobacteria and Proteobacteria phyla enrichment. These results suggesting an association between lung-microbial community diversity and CS responsiveness in severe asthma are further supported by more recent data showing a significant inverse correlation linking phylogenetic diversity in induced sputum specimens collected from patients with moderate-to-severe asthma and ICS dose with the use of both univariate and multivariate regression analyses [110].

#### 2.7.3. Effect of Treatment

Data regarding the effect of asthma treatment on lung microbiome are scarce in literature. In their study, after initial bronchoscopic sampling (EBs) for lung microbiome assessment at enrollment, Durack et al. randomized (2:1 ratio) 42 patients with mild well-controlled asthma, who had not received ICS previously, to receive a six-week course of a medium dose of ICS (250mcg fluticasone propionate twice daily) or placebo and repeated EBs post-treatment [62]. Despite limitations related to sample quantity insufficiency, the authors did not discern changes in bacterial burden and diversity after ICS treatment. However, they did find fluticasone-induced alterations in the relative abundance of certain taxa, namely an expansion of the *Microbacteriaceae* family and the *Moraxella* and *Neisseria* genera and a depletion of *Fusobacterium*, in those who responded to ICS treatment.

In another study, patients with both mild and more severe asthma were stratified according to the use of corticosteroids (inhaled and oral) [48]. Both those on ICS only and those on combined ICS and OCS treatment regimens exhibited significant alterations in EBs bacterial composition at the phylum, as well as the genus, level compared with corticosteroid-naïve asthma patients. These alterations comprised Proteobacteria enrichment and Bacteroidetes (specifically *Prevotella*) and Fusobacteria depletion in all corticosteroid groups, with decreased *Veillonella* and increased *Pseudomonas* abundance in those on ICS only and OCS, respectively. Although limited and centered exclusively on corticosteroids, these data clearly imply that asthma treatment may modify important compositional characteristics of the lung microbiome, including selection of potentially pathogenic species, with as yet unknown potential consequences. 

Interestingly, most recent studies have attempted to provide insights into the possible functional properties of lung microbiome in asthma. Durack et al. employed an algorithmic prediction model (PICRUSt) to infer lung bacterial metagenomic characteristics based on 16S-rRNA sequencing in patients with mild asthma and showed potentially increased expression of genes involved in pathways mediating the metabolism of amino acids and carbohydrates, particularly SCFAs, in these patients compared with healthy controls [62]. On the contrary, a relative reduction was predicted in the activation of LPS synthesis-specific processes. Using the same analytical method in severe asthmatic patients, Huang et al. managed to associate distinct metabolic (e.g., carbohydrate digestion, indole alkaloid biosynthesis) and immune (e.g., NOD-like and RIG-I-like receptor signaling) pathways with taxa (positively or negatively) correlated with specific phenotypical features of asthma, including body-mass index (BMI), asthma control assessed by Asthma Control Questionnaire (ACQ), corticosteroid responsiveness, and Th17-driven inflammation [104]. The authors concluded that concrete members of the asthmatic lung microbiota may be actively engaged in the pathogenetic mechanisms leading to the acquisition of different disease characteristics between asthmatics (i.e., phenotypes). Furthermore, Sverrild et al., again with the use of PICRUSt, showed different predicted functional profiles of lung microbiota associated with mild eosinophilic compared with non-eosinophilic asthma [107]. Clearly, further research taking advantage of the novel technologies in metagenomic analysis of lung microbiota is urgently needed to better describe the potential mechanistic role of lung microbiome alterations in asthma pathogenesis and disease phenotype/endotype determination. 

#### 2.7.4. Bronchial Hyperresponsiveness (BHR)

BHR to direct or indirect stimuli, in addition to airway inflammation, has long been considered inherent to asthma. However, neither its presence nor its severity is uniform or stable in all asthmatic individuals, and, thus, BHR may potentially constitute a contributing factor in asthma phenotyping [118]. BHR (indicated by metacholine PC20 concentrations) has been shown to correlate significantly with lung microbiota diversity in asthma, with greater bacterial diversity associated with lower metacholine PC20 concentrations [101]. In the same study, Proteobacteria comprised the majority of resident taxa correlated with greater BHR. Likewise, lung microbiota diversity was greater in those patients with asthma that exhibited the larger reductions in BHR following a course with clarithromycin [101].

#### 2.7.5. Lung Function

The potential impact of lung microbiome composition on lung function in patients with asthma has mainly been addressed by the study of Denner et al. [48]. The authors stratified their cohort of asthmatic patients (from across the range of disease severity) according to FEV_1_ and noticed a significantly decreased relative abundance of various bacterial phyla (Firmicutes, Bacteroidetes, and Actinobacteria) and genera (*Prevotella, Veillonella,* and *Gemella*) in the BAL fluid of those with the lowest FEV_1_ values (FEV_1_ < 60%). In contrast, *Lactobacillus* was found to be enriched in patients with the most severely impaired lung function. Similar positive correlations between the abundance of the *Bacteroidaceae* family or lung microbiota phylogenetic diversity and FEV_1_ have also been reported elsewhere [108,110]. Others have reached different conclusions with respect to taxa-specific associations with lung function in severe asthma, showing significantly reduced FEV_1_ levels in those patients with *M. catarrhalis, Haemophilus* sp, or *Streptococcus* sp predominance in induced sputa microbiota compared with others who had different taxa dominating the bacterial communities of their sputa [103]. 

#### 2.7.6. Obesity

Obesity has repeatedly been identified in cluster analyses of asthmatic populations as a key clinical feature discriminating a distinct subset of patients (mostly female) with adult onset, non-Th2 mediated, highly symptomatic and difficult-to-treat asthma from other asthma phenotypes [119,120,121]. Although the exact pathogenetic mechanisms linking obesity with asthma remain unknown, studies in obese asthmatics have shown improvements in BHR, asthma control, lung function, and inflammatory indices following bariatric surgery [122,123] or diet-induced [124,125] weight loss. 

In a cohort of patients with severe asthma, high BMI was significantly associated with a distinctive lung microbiota composition, mainly consisting of Bacteroidetes (including *Prevotella* species) and Firmicutes (e.g., *Clostridium* species) [104]. As expected, these obese asthmatic individuals presented less bronchial eosinophilic infiltration in EB specimens (non-eosinophilic asthma). It is noteworthy that bacterial taxa associated with obesity in this study exhibited distinct predicted functions, including activation of pathways involved in host immune response, such as NOD-like receptor signaling. These data suggest a potential role for lung microbiome in shaping the obesity-related asthma phenotype.

#### 2.7.7. Smoking Asthma

Although no significant difference has been reported in BAL bacterial communities composition between healthy smokers and non-smokers [42], an analysis of induced sputum microbiota profiles in patients with severe asthma demonstrated increased total bacterial diversity and relative abundance of Fusobacteria in ex-smokers compared with never smokers, while smoking intensity (assessed by pack–years of smoking) was directly associated with the prevalence of Actinobacteria [105]. These findings raise the possibility that smoking may be involved in shaping lung microbiome alterations observed in asthma.

### 2.8. Lung Microbiome and Asthma Exacerbations

**Figure 7:**
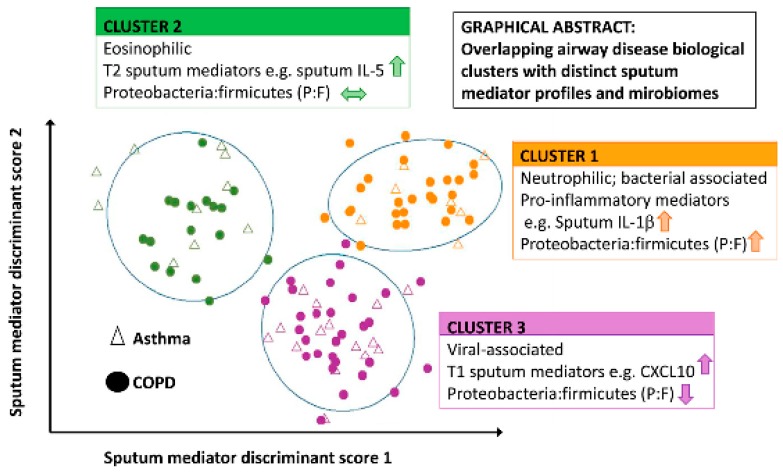
Exacerbation biologic clusters in asthmatic and COPD patients using the subjects’ discriminant scores. Adapted from ref. [130], Creative Commons Attribution License (CC BY).

The most common precipitating factor triggering asthma exacerbations is viral respiratory tract infections, particularly from rhinoviruses [126]. On the contrary, bacterial lung infections have not been shown to trigger asthma exacerbations but for in a minority of cases, with the possible exception of the self-limiting atypical bacterial infections caused by *Mycoplasma pneumoniae* and *Chlamydophila pneumoniae* for both of which a relative prevalence of 18% has been serologically detected during asthma attacks in adults and children, respectively [127]. On the other hand, considerably high rates of the potentially pathogenic Proteobacteria *Haemophilus influenzae* (the most prevalent), *Streptococcus pneumoniae*, and *Moraxella catarrhalis* have been detected by both culture-based and molecular techniques from upper- and lower-respiratory-tract samples of asthmatic children during exacerbation periods [128,129]. Moreover, a cluster analysis based on sputum cellular and mediator profiles in adult asthmatic patients during exacerbations has linked the predominance of different bacterial phyla in lung microbiota with specific clinical features and inflammatory markers [130] (see Figure 7). 

In order to provide an explanation for the above observations, a model of respiratory dysbiosis involving an altered lung microbiome and a dysregulated host immune response has been proposed to delineate the occurrence of asthma (and other chronic respiratory diseases) exacerbations [131]. According to this model, an acute stimulus, most commonly a viral infection, induces an immune-mediated inflammatory response, leading to a modification of local conditions in the airways, which may promote alteration of lung microbiota composition by favoring the predominance of specific previously underrepresented species and/or by predisposing to invasion by new potentially pathogenic strains. In turn, this deranged microbial growth may result in a further amplification of local airway inflammation, presumably via bacterial metabolite production and activation of signaling pathways mediated by the interaction between PAMPs and PRRs. Ultimately, a self-perpetuating vicious cycle arises, leading to the overwhelming inflammatory response that underlies asthma exacerbations. 

Indeed, there is considerable evidence suggesting that viral infections can increase susceptibility to bacterial growth and invasion. Viral infections may impair mucociliary clearance through diverse mechanisms, including increased mucous production, ciliary dyskinesia, and cilia depletion [132,133]. Moreover, rhinoviral and possibly other viral infections have been reported to facilitate bacterial transmigration by increasing airway epithelial barrier permeability in vitro, and this effect has been attributed to the loss of occludins, a major component of tight junctions connecting adjacent epithelial cells [134,135]. Respiratory viruses alter the nasopharyngeal microbiome and may be associated with a distinct microbial signature. In the study by Rosas-Salazar et al. [136], which mostly included children <6 months of age, nasopharyngeal microbiome of infants during HRV and RSV acute respiratory tract infections (ARIs) was largely dominated by Moraxella, Streptococcus, Corynebacterium, Haemophilus, and Dolosigranulum. In addition, there was a higher abundance of Staphylococcus and a trend toward a higher abundance of Haemophilus in RSV-positive infants, and in the overall bacterial composition between infants with HRV and RSV ARIs. Furthermore, Santee et al. [137] showed that previous history of acute sinusitis influences the composition of the nasopharyngeal microbiota, characterized by a depletion in relative abundance of specific taxa. Diminished diversity was associated with more frequent upper-respiratory infections. These, among many other published data, highlight the possibility that virus-specific compositional shifts in the nasopharyngeal microbiome contribute to worse outcomes after early-life ARIs.

Various viruses, including human rhinovirus (HRV), respiratory syncytial virus (RSV), and influenza virus, can augment the expression of adhesion molecules, such as ICAM-1, CEACAM-1, CEACAM-6, and PAFR, on the surface of epithelial cells, thus promoting invasion by certain bacterial species, including *H influenza, S pneumoniae*, and *M catarrhalis* [138,139,140]. Apart from the upregulation of host receptors, some viral structures appear to directly promote bacterial binding to host tissues [141,142]. 

Furthermore, viral infections may interfere with host immune system integrity and function and, consequently, increase susceptibility to bacterial colonization and infection. For instance, HRV infection has been shown to attenuate the inflammatory response to *H. influenzae* and delay bacterial elimination by decreasing TLR2 responsiveness to bacterial insult [143]. An almost complete disappearance of alveolar macrophages has been observed after influenza infection in a murine model [144] and HRV and RSV have been found to compromise phagocytosis and diminish pro-inflammatory cytokine release from alveolar macrophages in response to bacterial products [145,146]. Dendritic cell (DC) as well as CD4 and CD8 T-cell numbers and/or function may also be impaired after influenza or other viral infections with detrimental effects on host defense against bacteria [147]. Finally, viral respiratory infections induce changes in the lung microenvironment, such as increased temperature, nutrient availability, and cytokine and catecholamine levels, all of which are potential enhancers of bacterial virulence and immunogenicity [78,79,148]. The above mechanisms may act synergistically to elicit potentially significant alterations in the composition and diversity of lung microbiota, thus resulting through the dynamic microbiome–host immune system interplay, in the amplified and deregulated inflammatory response characterizing asthma exacerbations and even predispose to recurrent exacerbations. 

Conversely, there are data, albeit rather limited, suggesting that bacteria can influence susceptibility to viral infections. In this context, several experimental studies have provided evidence for a protective role of a healthy intact microbiome against influenza. Ichinohe et al. showed that mice with impaired microbiome as a result of pretreatment with antibiotics presented attenuated CD4 and CD8 T-cell responses and reduced antibody production following respiratory influenza virus infection [149]. Likewise, increased mortality has been reported in SPF mice intranasally infected with a lethal dose of influenza virus compared with non-SPF mice, and this finding could be replicated by priming mice with *Staphylococcus aureus*, used as a surrogate of upper-respiratory-tract commensal flora, before influenza virus inoculation [150]. TLR2 signaling and an M2 alveolar macrophage phenotype were essential for the relative insensitivity against influenza-induced lethal lung injury observed in this S aureus-primed murine model. Other studies focusing on the nasopharyngeal microbiota in children with RSV bronchiolitis have highlighted the potentially deleterious effects of a deranged Proteobacteria (*H influenzae*, *M catarrhalis*)-dominated microbiome on the risk of viral infection [151,152]. RSV infection occurrence and severity and pro-inflammatory cytokine concentrations were all found to be positively correlated with nasopharyngeal colonization with the potentially pathogenic *H. influenzae, M. catarrhalis,* and *Streptococcus species.* Similarly, *S. pneumoniae* colonization was associated with increased rates of seroconversion to human metapneumovirus in infants in an older study [153]. This viral infection predisposition conferred by the coexistence of potentially pathogenic bacteria could be the result of upregulation of viral entry receptors and/or impaired antiviral immune response induced by these bacteria. Such examples are the upregulation of the major HRV entry receptor ICAM-1 by *H. influenza* in airway epithelial cells [154] or the reduction of TLR3 expression and IFN secretion in bronchial epithelial cells infected with *M catarrhalis* [155]. 

Collectively, these data strengthen the hypothesis that lung microbiome alterations observed in asthma patients are actively involved in the pathogenesis of disease exacerbations and may represent a potential therapeutic target in asthma exacerbations management. 

### 2.9. Therapeutic Implications of Microbiome Manipulation

Based on the above, the proposed role of human microbiome in shaping both asthma susceptibility and phenotypes renders exogenous manipulation of microbiome a potentially attractive therapeutic strategy for both asthma prevention and treatment. Interventions in microbiome composition have previously been recommended as putatively effective approaches in three areas of asthma management: (1) prevention of asthma development during early life by favoring factors that promote immune tolerance to allergens and minimizing those that predispose to the emergence of atopy; (2) management of Th2-low asthma phenotypes/endotype, particularly neutrophilic asthma with frequent exacerbations; and (3) reverse of CS resistance or prevention of its emergence [156]. As yet, potential interventions applied for these purposes comprise lifestyle measures, vaccinations, and pharmacological treatment, including (1) probiotics, (2) prebiotics, and (3) antibiotics. It must be emphasized that, until now, only scarce data are available for the exact impact of these interventions on human microbiome composition and function, and even less is known about their presumed ability to ameliorate clinically meaningful outcomes in asthmatic patients. Furthermore, the effects of medications currently used in asthma treatment, including CS and bronchodilators, on microbiome remain mostly undetermined, and microbiome manipulations could ideally enhance beneficial actions and/or minimize side effects possibly induced by these agents. 

Theoretically, any environmental exposure capable of modifying the composition of human microbiome during the dynamic period of its acquisition and maturation starting in utero and extending to the first few years of life may influence the likelihood of subsequently developing allergic asthma. This means that there is a so-called ‘window of opportunity’ estimated to operate within the first 100 days of life, during which lifestyle interventions, as well as exogenously administered microbial agents, could promote the formation of a tolerogenic microbiome, thus limiting the risk of allergic asthma [157]. For instance, raw cow milk consumption and vitamin D and omega-3 fatty acids supplementation during pregnancy have all been shown to diminish the risk of asthma in the offspring, presumably by affecting the microbiome synthesis of the child [158]. Avoidance of unnecessary maternal antibiotic exposure is another potentially beneficial pre-birth measure linked with microbiome [89]. As previously mentioned, vaginal delivery, breastfeeding, reasonable antibiotic use, pet ownership, exposure to ‘unhygienic’ traditional farming environments, and high-fiber dietary content in infancy have, more or less consistently, been associated with favorable effects on human body microbiota composition and function (especially in the gut) and a reduced risk of allergic asthma development later in life. However, it must be stressed that available evidence is generally inconclusive and probably only a few of these associations can be considered definite. Moreover, implementation of some or even all of these measures may be practically unattainable for various reasons.

To overcome these limitations, more “interventional” approaches have been proposed. Probiotics are live microorganisms (bacteria or fungi) that may favorably affect the host health, whereas prebiotics are defined as non-digestible compounds that, after processing by intestinal microbiota, promote the expansion and/or activation of beneficial commensals. Symbiotics refer to preparations containing both probiotics and prebiotics [159]. A multitude of orally administered probiotics have been shown to attenuate allergic airway inflammation in mice [160,161,162,163]. As described above, high-fiber diets and SCFAs administration (which are essentially prebiotics) have also been found to mitigate inflammatory changes in experimental models of allergic airway disease [99,100]. The potential effect of these biologic agents with microbiome-modifying capacity on allergic asthma has further been studied in several randomized placebo-controlled trials (RCTs). Daily supplementation of asthmatic school children with the probiotic *Lactobacillus gasseri* A5 or a mixture of *Lactobacillus acidophilus*, *Bifidobacterium bifidum*, and *Lactobacillus delbrueckii* for two to three months have both been shown to improve asthma symptoms and lung function [164,165,166,167,168]. Similarly, a symbiotic preparation consisting of short-chain galacto-oligosaccharides, long-chain fructo-oligosaccharides, and the *Bifidobacterium breve* M-16V increased PEF and reduced serum IL-5 in an adult cohort of allergic asthma [168]. However, most [167,168,169], albeit not all [170], meta-analyses performed to date have failed to demonstrate a beneficial effect of preventive probiotic supplementation to mothers during pregnancy and/or infants during the first year of life on asthma risk. Further adequately powered and well-designed RCTs are warranted to clarify the role of probiotics/prebiotics on asthma prevention and treatment. 

While the possible propitious effects of orally administered probiotics/prebiotics on asthma are thought to be derived by favorable alterations in the gut microbiota and the gut–lung axis, a more targeted approach involving direct interventions to lung microbiome via inhalational delivery of such agents remains relatively unexplored. In mice, intranasal administration of *Acinetobacter Iwoffii, Lactococcus lactis,* or *Staphylococcus sciuri*, all of which had previously been isolated from cowsheds in the context of farming asthma studies, have been shown to hinder eosinophilic airway inflammation induced by ovalbumin sensitization and challenge [2,171]. Similar findings have also been reported by another group for *Escherichia coli* administered via inhalation in the same murine model of allergic airway inflammation [172]. In all cases, allergy protection conferred by inhaled bacteria was associated with modifications in DC activation, leading to altered T-cell responses [172,173,174], in line with the previously presented observations on SCFAs administration. However, the effects of inhaled probiotic treatments on lung microbiota structure and function have not been addressed. In another experimental study assessing the potential influence of the administration route on the protective role of probiotics, *Lactobacillus paracasei* more efficiently suppressed eosinophilic inflammation when administered intranasally rather than via a feeding tube [175]. These results might suggest superior efficacy of microbiome-modifying therapeutic interventions, probiotics, and probably others (e.g., antibiotics) [176], when applied locally to the respiratory tract as compared to oral administration, which additionally may affect other organs. As yet, no human data are available for inhaled probiotic treatment in asthma.

The proposed role of human microbiome on shaping asthma phenotypes and exacerbation susceptibility, as analyzed above, may form the conceptual basis for the use of antibiotics as a means for therapeutic manipulation of the microbiome, aiming at the restoration of a symbiotic state between the host and the resident microbiota through the suppression of overgrown detrimental taxa and the foster of beneficial ones. This holds especially true for the Th2-low, CS-insensitive, exacerbation prone, severe neutrophilic asthma phenotype, the treatment options for which are particularly limited. As Proteobacteria-dominated changes in lung microbiome have quite consistently been identified in relation with this phenotype, with *Haemophilus influenzae* and *Moraxella catarrhalis* being the most prominent species involved, these taxa could probably be considered the primary targets of microbiome-modifying antibiotic treatment in neutrophilic asthma. The anticipated gradual advent of non-culture-based methods for lung microbiota identification in clinical practice may allow more personalized approaches in antibiotic targeting of dysbiotic lung bacteria in the future.

Both in asthma and in a growing list of other respiratory diseases, macrolides have become the subject of much attention due to the fact that they combine well-known antimicrobial activity along with multiform immunomodulatory properties, including attenuation of neutrophil chemotaxis, inhibition of biofilm formation, reduction of mucus hypersecretion, and even downregulation of viral entry receptors with amplification of viral infection-induced IFN production [177,178,179]. Overall, clinical trials of macrolides in asthma, most of which in patients with severe uncontrolled asthma, have yielded conflicting results. However, in a pre-specified subgroup analysis of the AZISAST multicenter randomized placebo-controlled trial, it was shown that a six-month course of azithromycin, given at a dose of 250 mg, three times a week, in patients with severe asthma and frequent exacerbation, significantly reduced a composite primary outcome comprising the rates of severe exacerbations and lower respiratory-tract infections requiring antibiotics in the non-eosinophilic asthma subgroup of patients [180]. More recently, the larger AMAZES randomized controlled trial (RCT) reported significantly decreased rates of exacerbations, along with improved asthma-related quality of life in patients with uncontrolled asthma despite therapy with medium-to-high doses of ICS plus LABA who received a higher dose of azithromycin (500 mg three times per week) for 12 months [181]. Interestingly, in this trial, azithromycin appeared equally effective in all studied subgroups, including both those of eosinophilic and non-eosinophilic asthma. Although these beneficial effects of azithromycin (and perhaps other macrolides) on asthma exacerbations could be attributed to the aforementioned immunomodulatory anti-inflammatory properties of macrolides, there are data showing alterations of microbiota composition following treatment with azithromycin. In a research letter published in 2014, Slater et al. described the longitudinal effects of six weeks of daily therapy with 250 mg azithromycin on lung microbiota characteristics, using bronchoscopic washings for sampling and DNA pyrosequencing analysis in five patients with moderate-to-severe asthma [182]. The study reported a reduction of lung microbiota diversity post-treatment, accompanied by an increased relative abundance of *Anaerococcus* and a decreased recognition of the potentially pathogenic genera of *Pseudomonas*, *Staphylococcus,* and *Haemophilus*. Furthermore, in a bacteriological sub-study of the AZISAST trial, azithromycin administration was associated with post-treatment changes in the microbiota composition of the oropharynx compared with both pretreatment status and control patients receiving placebo [183]. At the phylum level, Firmicutes increased and Fusobacteria decreased in oropharyngeal swabs derived from eight severe asthma patients after a six-month course of azithromycin. This finding corresponded to an increased abundance of *Streptococcus salivarius*, with a parallel reduction of *Leptotrichia wadei* at the species level. Of notice, in half of the washout samples collected one month following completion of azithromycin treatment, the microbiota composition was almost identical to the pretreatment oropharyngeal flora of the same patient, indicating a possibility for original microbiome recovery after cessation of antibiotics administration and a consequent requirement for long-term treatment.

Admittedly, there are considerable limitations in the long-term use of antibiotics as a therapeutic tool for favorable manipulations of the host microbiome. Apart from well-founded concerns with respect to potential adverse effects (sometimes irreversible or life threatening) and emergence of antimicrobial resistance arising from long-term antibiotic regimens, the lack of specificity in the elimination of resident bacteria does not allow currently available antimicrobial agents to eradicate exclusively dysbiotic members of the lung bacterial community while sparing beneficial commensals, and may, in fact, produce the opposite results. As previously noted, maternal and early life antibiotic usage has been associated with an increased risk of asthma development during childhood. The detrimental effects of antibiotic use (and not necessarily long-term) on gut microbiome are firmly established and involve the depletion of normal biodiversity and the overgrowth of potentially harmful bacterial strains, leading to dysbiosis. Occasionally, this can be expressed clinically in the form of antibiotic-associated diarrhea and pseudomembranous colitis. Such antibiotics-associated hazards (i.e., selection of potentially pathogenic microbiota) are also pertinent to the lung microbiome, and broad-spectrum antibiotic use may lead from a state of respiratory dysbiosis to another, each time dominated by different and probably more-resistant strains. Despite potentially abolishing systemic adverse effects, inhaled antibiotics are probably subject to these same limitations and cannot be considered as an ideal alternative to systemically administered agents. 

Novel approaches to microbiome-modifying therapies have focused on the manipulation of metabolites known to be produced or processed by the (gut) microbiota and to act on the host [184,185]. Metabolite-targeting molecules are under development. Fecal transplantation has successfully been used as a means of gut microbiota manipulation in several intestinal disorders [186]. Its potential role in allergic and respiratory diseases remains unexplored.

The endeavor to therapeutically manipulate human microbiome is clearly still in its infancy, and, by all means, intensive innovative research applying to all stages of the treatment discovery and development pipeline is mandatory before any of these interventions could be incorporated into clinical practice guideline recommendations.

## 3. Conclusions

Studies conducted over the last two decades have dramatically changed our perspective on LRT microbiology, with the use of culture-independent molecular techniques. It is now universally accepted that the lung is far from a sterile organ. It has convincingly been shown that, in asthma, lung microbiome undergoes significant alterations in terms of diversity and composition, with certain species outgrowing others, functionally leading to a presumable state of respiratory dysbiosis. Many of these alterations have been associated with specific phenotypic features of asthma, including disease exacerbations, and aspects of this dysbiosis may represent the missing link in the pathogenesis of some expressions of the asthmatic disease, perhaps, in particular, the neutrophilic inflammatory phenotype. A considerably large body of evidence arising from both experimental and epidemiological studies is now available, suggesting that early life environmental exposures affecting the gut microbiome structure may be involved in shaping susceptibility for asthma development later in life by modifying microbiota-derived factors thought to be actively involved in the configuration of the gut–lung axis, such as the SCFAs. Given its proposed roles in influencing the risk for asthma development, as well as the phenotypic expression of an established disease, microbiome has emerged as a potential therapeutic target in asthma. As yet, interventions potentially modifying the microbiome have not clearly been shown to be efficacious in preventing and/or treating asthma, with few exceptions. 

More accurate delineation of lung microbiota structural and functional characteristics, both in health and in asthma, remains an unmet need in the microbiome research. Larger longitudinal studies applying standardized sampling methods in well-characterized asthmatic cohorts are indispensable in this regard. Unquestionably, the impending widespread application of the novel “-omics” technologies can be expected to provide invaluable insights into the functional effects of lung microbiome and their presumable role in shaping asthma predisposition and phenotypes. Advancing our understanding on the mechanistic links between human microbiome and asthma will hopefully culminate in the discovery of novel therapeutic interventions targeting specific aspects of respiratory dysbiosis.
